# O-GlcNAcylation of SIRT1 Protects against Cold Stress-Induced Skeletal Muscle Damage via Amelioration of Mitochondrial Homeostasis

**DOI:** 10.3390/ijms232314520

**Published:** 2022-11-22

**Authors:** Yu Cao, Meng Zhang, Ye Li, Jingjing Lu, Wanhui Zhou, Xiaoshuang Li, Hao Shi, Bin Xu, Shize Li

**Affiliations:** 1 College of Animal Science and Veterinary Medicine, Heilongjiang Bayi Agricultural University, Daqing 163319, China; 2 Sheep Disease Laboratory, Branch of Animal Husbandry and Veterinary of Heilongjiang Academy of Agricultural Sciences, Qiqihar 161005, China; 3 Department of Animal and Poultry Sciences, Virginia Polytechnic Institute and State University, Blacksburg, VA 24061, USA

**Keywords:** cold stress, skeletal muscle, metabolic homeostasis imbalance, SIRT1, O-GlcNAcylation

## Abstract

Cold stress disturbs cellular metabolic and energy homeostasis, which is one of the causes of stress-induced illnesses. O-GlcNAcylation is a nutrient-sensing pathway involved in a myriad of cellular processes. It plays a key role in metabolic homeostasis. Nevertheless, a specific sensing mechanism linking skeletal muscle to O-GlcNAcylation in cold stress is unknown. In this study, O-GlcNAcylation of SIRT1 was targeted to explore the mechanism of skeletal muscle adaptation to cold stress. *Ogt* mKO aggravated skeletal muscle fibrosis induced by cold stress. At the same time, *Ogt* gene deletion accelerated the homeostasis imbalance and oxidative stress of skeletal muscle mitochondria induced by cold stress. In vitro results showed that inhibition of SIRT1’s O-GlcNAcylation accelerated mild hypothermia induced mitochondrial homeostasis in mouse myogenic cells (C2C12 cells). However, overexpression of SIRT1’s O-GlcNAcylation improved the above phenomena. Thus, these results reveal a protective role of OGT-SIRT1 in skeletal muscle’s adaptation to cold stress, and our findings will provide new avenues to combat stress-induced diseases.

## 1. Introduction

Over the past decade, cold stress (CS) has become an important research topic in stress biology. Exposure to cold is a common form of stress, when the body experiences cold stress, the stability of the body’s internal environment is disrupted, which can lead to biological damage, which can interfere with energy metabolism and cause endogenous or secondary illnesses [1,2,3,4,5,6]. Studies have shown that cold exposure induces the production of several cytokines and/or various factors that might increase cancer risk [7]. Additionally, cold exposure stress causes hypothermia, cognitive impairment, liver injury, and cardiovascular diseases, thereby increasing morbidity and mortality [8]. Additionally, other research suggests that functional hyperthyroidism, lower urinary tract symptoms, hippocampal neurotransmitter disorder and neuroinflammation are associated with cold stress [9,10,11]. In addition, cold stress can destroy the equilibrium between oxidants and antioxidants and induce excessive production of reactive oxygen species (ROS) in the body, thereby causing oxidative damage to biological macromolecules [12]. Therefore, it is of great significance to explore the physiological function changes of the body during cold stress to reveal the cold adaptation mechanism, improve cold resistance, and prevent cold damage.

O-linked-N-acetylglucosaminylation (O-GlcNAcylation) is a dynamic post-translational modification of numerous proteins in the nucleus, cytoplasm, and mitochondria that is linked to energy production and cellular metabolism [13,14]. O-GlcNAcylation cycling is facilitated by only two enzymes: O-GlcNAc transferase (OGT), which catalyzes the addition of GlcNAc from a high-energy donor substrate to the hydroxyl groups of serine (Ser) and threonine (Thr) residues of the target protein, while O-GlcNAcase (OGA) catalyzes the removal of GlcNAc [15]. O-GlcNAcylation could regulate signaling, transcription, translation, division, metabolism, and stress sensitivity in all cells [16]. Deregulation of O-GlcNAcylation has been reported to be associated with various human diseases, such as cancer, diabetes, neurodegenerative diseases, cardiovascular diseases, and diabetic nephropathy [17]. Disturbances in the regulation of cellular volume may contribute to disease in settings of chronically elevated O-GlcNAcylation, including diabetic nephropathy [18]. Since O-GlcNAcylation is regarded as a nutrient-sensing pathway, it is not surprising that many mitochondrial proteins are O-GlcNAcylated. For example, several mitochondrial proteins involved with the electron transport chain, including subunit NDUFA9 of complex I, subunit cores 1 and 2 of complex III, and mitochondrial DNA-encoded subunit I of complex IV, and proteins involved with the tricarboxylic acid cycle, including succinyl-coasynthetase and oxoglutarate dehydrogenase complex, are O-GlcNAcylated [19,20]. O-GlcNAcylation of mitochondrial proteins play an essential role in glucose homeostasis in response to CS [21]. In addition, the O-GlcNAc/Akt pathway is reported to regulate glucose metabolism and reduce apoptosis in the pig liver in response to acute CS [22]. In brown adipose tissue, O-GlcNAcylation plays a pivotal role in CS-induced mitochondrial biogenesis and thermogenesis [23]. Acute CS is also reported to increase O-GlcNAcylation levels in mouse SM cells while reducing apoptosis and autophagy [24]. In muscle tissue, levated muscle O-GlcNAc levels paralleled insulin resistance and type 2 diabetes in humans. Moreover, OGT-mediated signaling is necessary for proper skeletal muscle metabolism and whole-body energy homeostasis, and data highlights O-GlcNAcylation as a potential target for ameliorating metabolic disorders [25]. Together, these findings suggest that O-GlcNAcylation may serve to promote cell survival and balance cellular metabolism in response to CS.

Silent information regulator factor 2-related enzyme 1 (Sirtuin 1, SIRT1) is a nicotinamide adenine dinucleotide (NAD^+^)-dependent deacetylase involved in the regulation of a wide range of biological processes, including cell senescence, energy balance, and oxidative stress (OS) [26,27]. NAD^+^ and NADH are energy sources for the electron transport chain in mitochondria, and NAD^+^ is the substrate of SIRT1, which plays an important role in the regulation of mitochondrial oxidative phosphorylation [28]. In addition, SIRT1 activation is reported to cause the demise or turnover of damaged mitochondria, potentially through mitophagy [29,30,31,32]. Hence, SIRT1 has an important role in the maintenance of mitochondrial health [33]. Furthermore, SIRT1 has been established as a critical regulator of autophagy, which is broadly viewed as a protective mechanism against stress and cell death [34,35,36]. Upstream, O-GlcNAcylation of SIRT1 is elevated during genotoxic, oxidative, and metabolic stress, and increases deacetylase activity to protect cells against stress-induced apoptosis [37]. Downstream, SIRT1 has a large number of targets, including members of the Forkhead box class O family of proteins (FoxOs). SIRT1 regulates the activity of FoxOs, which in turn, modulates the activity of SIRT1 [38]. FoxOs influence a wide range of targets, including apoptosis and autophagy genes, anti-oxidative enzymes, cell cycle arrest genes, and metabolic and immune regulators [39,40]. FoxOs also regulate two main proteolytic systems: the ubiquitin-proteasome system and the autophagy-lysosome system, including mitophagy [41]. Given the multifaceted functions of FoxOs, it is reasonable to speculate that SIRT1-induced modulation of FoxO activity could influence mitochondrial function via gene expression in the nucleus and retrograde signaling from the mitochondria to the nucleus. Together, these findings suggest that SIRT1 and O-GlcNAcylation participate in maintaining cellular homeostasis in response to stressful conditions.

Skeletal muscle (SM) is the largest repository of proteins in all animals. The growth of SM is a complex molecular process controlled by various signal transduction pathways, regulatory factors, and genes [42]. Diseases of the SM are associated with significant changes to metabolic pathways and homeostasis [43,44]. Energy metabolism in the mitochondria is important to maintain the homeostasis of SM cells. As an energy converter, mitochondria produce adenosine triphosphate (ATP) through aerobic respiration to supply cellular energy and regulate mitochondrial homeostasis in response to stress [45,46,47]. Homeostasis of SM is linked to the number of mitochondria in SM fibers and mitochondrial respiration capacity. Mitochondria exhibit remarkable plasticity by adapting their volume, structure, and function in response to chronic exercise, disuse, aging, and disease [48]. Cellular mitophagy is a cellular process that selectively removes aged and damaged mitochondria, which is important for cellular homeostasis [49,50,51]. Excessive mitochondrial fusion can enhance mitophagy, thereby reducing mitochondrial mass and ATP production [52]. Fibrosis of SM is a hallmark of muscular dystrophies, aging, and severe muscle injuries [53]. During the aging process, decreased expression of Mitofusin 2 and accumulation of aberrant mitochondria contribute to sarcopenia [54,55]. However, the effects of CS on SM and the role of O-GlcNAcylation in this process remain unclear. In this research, SIRT1’s O-GlcNAcylation was targeted to explore the mechanisms underlying the adaptation of SM cells to acute CS.

## 2. Results

### 2.1. Ogt mKO Accelerated CS-Induced Damage to SM Cells

**Figure 1:**
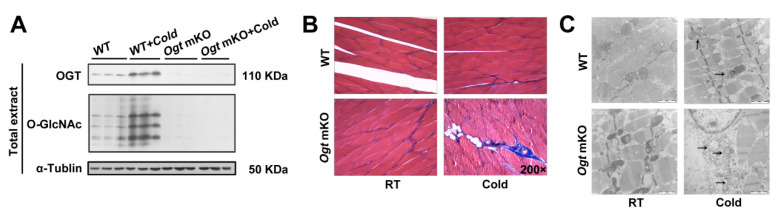
OGT mKO exacerbated CS-induced SM and mitochondrial damage. (**A**) Western blotting analysis using antibodies against OGT and O-GlcNAc. Tubulin served as a loading control. Expression of autophagy-related proteins in SM total extract and mitophagy-related proteins in mitochondrial extract. After CS treatment, fresh SM tissues of four groups (WT group, WT + cold group, *Ogt* mKO group, *Ogt* mKO + cold group) were collected, sectioned, and stained with (**B**) Masson’s trichrome and (**C**) Mouse SM tissue was sliced into ultrathin sections and observed under a transmission electron microscope. The arrows represent damaged mitochondria.

OGT was specifically knocked out in SM cells using a human SM α-actin-driven Cre/LoxP system because O-GlcNAcylation plays an important role in modulating cellular metabolic homeostasis [56,57,58]. O-GlcNAcylation and mKO of OGT were confirmed using antibodies against O-GlcNAc and OGT (Figure 1A). In response to acute CS, OGT and O-GlcNAcylated proteins were significantly upregulated (Figure 1A). Acute CS affects a wide range of biochemical processes, particularly metabolism, and SM is a major metabolic tissue in mammals. To study the effects of acute CS on SM, tissue sections were stained for markers of fibrosis (Figure 1B), which were increased in response to CS. Notably, OGT deficiency accelerated fibrosis of SM in response to CS. In addition, CS led to changes in mitochondrial morphology, which were comparatively more pronounced in *Ogt* mKO mice (Figure 1C). These findings indicate that CS damaged the organelles of SM cells, especially in the mitochondria, which was exacerbated by OGT deficiency.

### 2.2. OGT Deficiency Exacerbated Mitophagy and ROS in Response to CS

**Figure 2:**
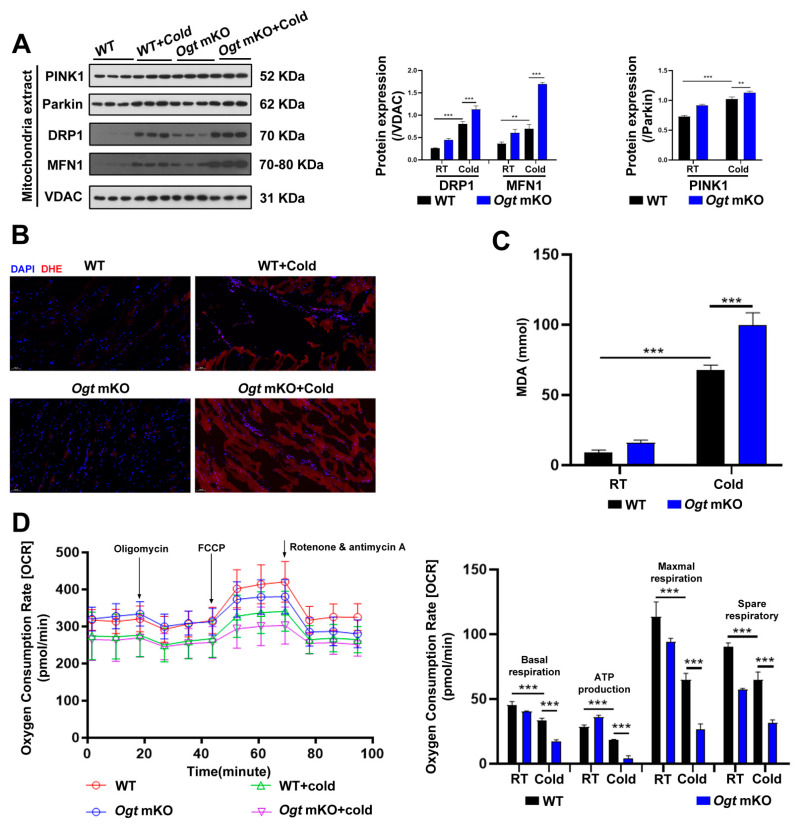
OGT mKO exacerbated CS-induced mitophagy. (**A**) After CS treatment, fresh SM tissues of four groups (WT group, WT + cold group, *Ogt* mKO group, *Ogt* mKO + cold group) were collected and prepared for Western blotting analysis using antibodies against PINK1, Parkin, DRP1, MFN1, and VDAC. (**B**) Fresh SM tissue from each group was collected, sectioned, and stained with DHE dye for ROS. (**C**) After CS treatment, fresh SM tissue was collected to detect the content of MDA. (**D**) Fresh SM tissue was prepared into ultrathin sections and mitochondrial function (i.e., basal respiration, ATP production, maximum respiration, and reserve respiratory capacity) was assessed with the cellular mitochondrial pressure test. All results contain 3 replicates per group (n = 3/group). Data are presented as means ± sd, and were analyzed by two-way ANOVA. **p < 0.01, *** *p* < 0.001.

In response to CS, mitochondria were damaged (Figure 1C). In the present study, the effects of CS on mitophagy in SM were assessed. The mitophagy markers PINK1 and Parkin, a ubiquitin ligase involved in the degradation of damaged mitochondria [59], which are involved in the degradation of damaged mitochondria, were increased in SM in response to CS and comparatively further increased in *Ogt* mKO mice (Figure 2A). In addition to the mitochondrial network’s maintenance, the expression levels of the mitochondrial fission marker DRP1 and the fusion marker MFN1 were examined, and both markers showed the same patterns as PINK1 and Parkin (Figure 2A). The accumulation of ROS leads to OS and mitochondrial dysfunction, which can result in organelle autophagy and cell apoptosis [60]. Therefore, markers of OS and apoptosis were measured in SM cells. The results showed that the content of ROS and MDA, end-products of peroxidation of polyunsaturated fatty acids, had increased dramatically in response to CS, particularly in *Ogt* mKO SM cells (Figure 2B,C). Then, the mitochondrial stress test was performed to assess mitochondrial function. The results showed that mitochondrial basal respiration, ATP production, maximal respiration, and reserve respiratory capacity were significantly downregulated in SM cells in response to acute SC and mKO of OGT further compromised mitochondrial function (Figure 2D). Together, these data suggest that CS induces mitophagy and ROS-associated damage to the mitochondria.

### 2.3. CS Enhanced Protein Acetylation in SM Cells via Inhibition of SIRT1

**Figure 3:**
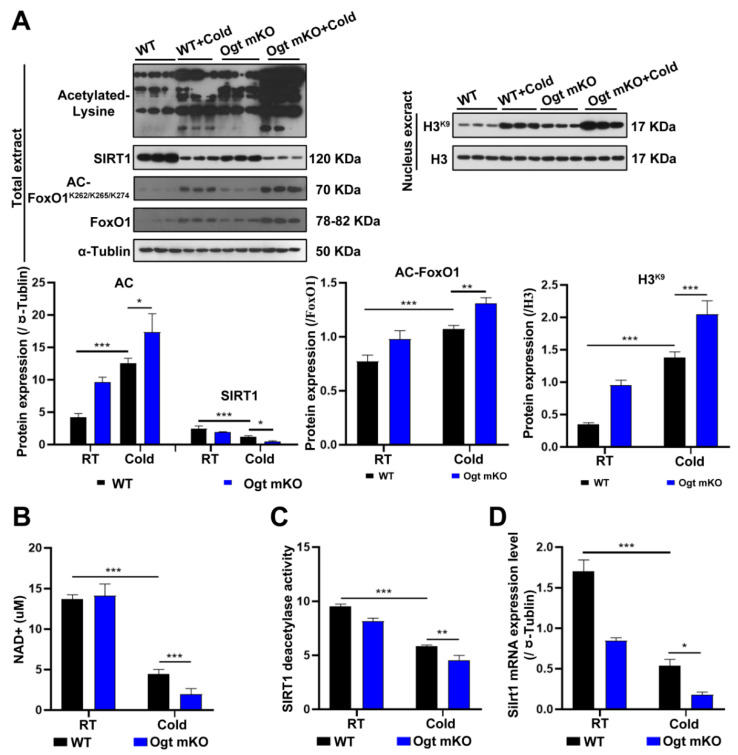
CS enhances protein acetylation via downregulation of SIRT1. (**A**) After CS treatment, fresh SM tissues of four groups (WT group, WT + cold group, *Ogt* mKO group, *Ogt* mKO + cold group) were collected and prepared for Western blotting analysis using antibodies against acetyl-Lys, SIRT1, acetyl-FoxO1, FoxO1, and tubulin. Expression of H3^K9^ and H3 in the nuclear extract of SM cells. (**B**–**D**) Fresh SM tissue was collected to detect (**B**) NAD^+^ content, (**C**) SIRT1 activity, and (**D**) *Sirt*1 mRNA expression. All results contain 3 replicates per group (n = 3/group). Data are presented as means ± sd and were analyzed by two-way ANOVA. * *p* < 0.05, ** *p* < 0.01, *** *p* < 0.001.

Since acetylation and deacetylation are involved in the initiation and selectivity of autophagy [61], the expression levels of acetylated proteins in SM cells were measured. The results showed that acetylated proteins in SM cells were significantly upregulated in response to CS, particularly in *Ogt* mKO C2C12 cells (Figure 3A). Since SIRT1 can deacetylate histones and histone-modifying enzymes [62], the abundance of SIRT1 and the acetylation status of histone H3 at Lys9 were investigated. The results showed that SIRT1 expression was significantly downregulated, while H3 acetylation at Lys9 was upregulated (Figure 3A). Since both OS and acetylation regulate FoxO1 activity [63], the expression levels of FoxO1 and acetylation status at Lys262, 265, and 274, which were upregulated by CS, were assessed (Figure 3A). The results showed that the SIRT1 substrate NAD^+^, SIRT1 deacetylase activity, and SIRT1 mRNA levels were all significantly decreased (Figure 3B–D). Together, these data suggest that acute CS increases acetylation of SM proteins via downregulation of SIRT1, which triggers autophagy and mitophagy.

### 2.4. MHT Restored the In Vivo Phenotype of C2C12 Cells

Hypothermia is defined as a decrease in body temperature to less than 35 °C. Hypothermia is classified as mild (32–35 °C), moderate (28–32 °C), severe (25–28 °C), and profound (<24 °C) based on severity [64]. Given that MHT affects cell growth and survival, C2C12 cells were cooled to 32 °C for 3, 6, and 9 h. The results showed that MHT induced autophagy and mitophagy and upregulated O-GlcNAcylation in C2C12 cells, similar to the response to CS in vivo (Supplementary Figure S1A,B). Therefore, in the following experiments, MHT was applied for 3 h.

### 2.5. O-GlcNAcylation Inhibition Increased Autophagy and Mitophagy in C2C12 Cells Exposed to MHT

**Figure 4:**
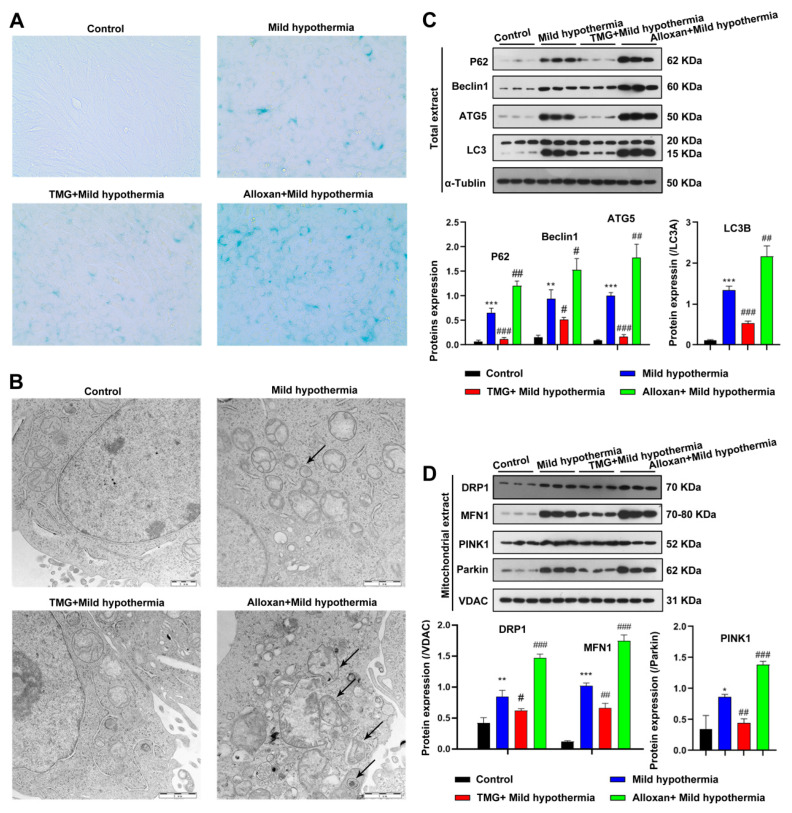
MHT induces autophagy and mitophagy in C2C12 cells. C2C12 cells were collected and prepared into ultrathin sections after 3 h of exposure to 32 °C, which were (**A**) stained for β-galactosidase and (**B**) the structure was examined using transmission electron microscopy. (**C**,**D**) Autophagy and mitophagy-related protein expression level in Total or mitochondrial extract. All results contain 3 replicates per group (n = 3/group). Data are presented as means ± sd and were analyzed by one-way ANOVA. * compared to control, * *p* < 0.05, ** *p* < 0.01, *** *p* < 0.001; # compared to mild hypothermia group, # *p* < 0.05, ## *p* < 0.01, ### *p* < 0.001.

To gain a mechanistic insight into O-GlcNAcylation in response to CS, C2C12 cells were employed as an in vitro model. In brief, C2C12 cells were exposed to MHT with the OGT inhibitor alloxan or the OGA inhibitor thiamet G to decrease or enhance O-GlcNAcylation, respectively [65]. Because OS cause premature aging of cells [66,67], the biomarker senescence-associated beta-galactosidase (SA-β-gal) activity was measured. The results showed that MHT led to an accumulation of SA-β-gal, consistent with the in vivo mouse model (Figure 4A). CS-induced aging was enhanced by inhibition of OGT and ameliorated by inhibition of OGA (Figure 4A). Furthermore, CS induced disorganization of the mitochondria of C2C12 cells (Figure 4B). In addition, MHT enhanced the expression levels of biomarkers of autophagy and mitophagy. Mitochondrial defects were exacerbated by inhibition of OGT and reversed by inhibition of OGA (Figure 4C,D). Taken together, these data suggest that O-GlcNAcylation protects cells from MHT.

### 2.6. MHT Disrupted Mitochondrial Homeostasis in C2C12 Cells

**Figure 5:**
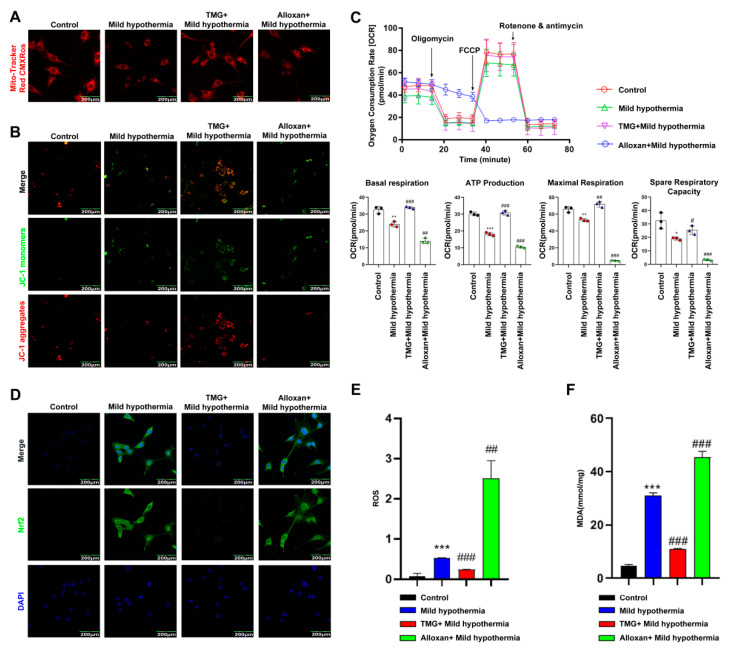
MHT compromises mitochondrial function. Mouse primary SM cells were isolated and cultured. After 3 h of MHT exposure at 32 °C, primary SM cells were collected for functional analysis. (**A**) MitoTracker™ Red CMXRos staining of C2C12 cells. (**B**) JC-1 staining of C2C12 cells. (**C**) Mitochondrial function (i.e., basal respiration, ATP production, maximum respiration, and reserve respiratory capacity) was assessed with the cellular mitochondrial pressure test. (**D**) Nrf2 immunocytochemistry staining in C2C12 cells. (**E**) Flow cytometry analysis of ROS production in C2C12 cells. (**F**) MDA content. All results contain 3 replicates per group (n = 3/group). Data are presented as means ± sd and were analyzed by one-way ANOVA. * compared to control, * *p* < 0.05, ** *p* < 0.01, *** *p* < 0.001; # compared to mild hypothermia group, # *p* < 0.05, ## *p* < 0.01, ### *p* < 0.001.

The MitoTracker™ Red CMXRos was used to determine the effect of MHT on the mitochondria of C2C12 cells. The results showed that the number of mitochondria was reduced in C2C12 cells in response to CS. Inhibition of OGT with alloxan exacerbated this effect, while inhibition of OGA with thiamet G had the opposite effect (Figure 5A). JC-1 is a fluorescence probe widely used as a sensitive marker to detect mitochondrial membrane potential [68]. When mitochondrial membrane potential is high, JC-1 aggregates in the mitochondrial matrix and forms J-aggregates, which produce red fluorescence. The results showed that the mitochondrial membrane potential of C2C12 cells decreased in response to MHT. Inhibition of OGT further lowered mitochondrial membrane potential, whereas inhibition of OGA reversed this effect (Figure 5B). Moreover, MHT significantly downregulated basal respiration, ATP production, maximum respiration, and reserve respiratory capacity of isolated mitochondria. Inhibition of OGT further damaged mitochondrial function, whereas inhibition of OGA reversed this effect (Figure 5C). Since Nrf2 has been shown to regulate defense against OS [69], the cells were stained with antibodies against Nrf2. The results showed that MHT induced Nrf2 expression. Inhibition of OGT enhanced Nrf2 expression, whereas inhibition of OGA reversed this effect (Figure 5D). To assess ROS production, C2C12 cells were loaded with dichlorodihydrofluorescein diacetate for detection of intracellular hydrogen peroxide and OS [70]. The flow cytometry results showed that MHT increased ROS production, which was further enhanced by inhibition of OGT and blocked by inhibition of OGA (Figure 5E). MDA is a final product of polyunsaturated fatty acid peroxidation and, thus, a marker of OS [71]. In this study, MHT induced overproduction of MDA, which was further increased by inhibition of OGT, while inhibition of OGA reversed this process (Figure 5F).

### 2.7. MHT Inhibited SIRT1 O-GlcNAcylation in C2C12 Cells

**Figure 6:**
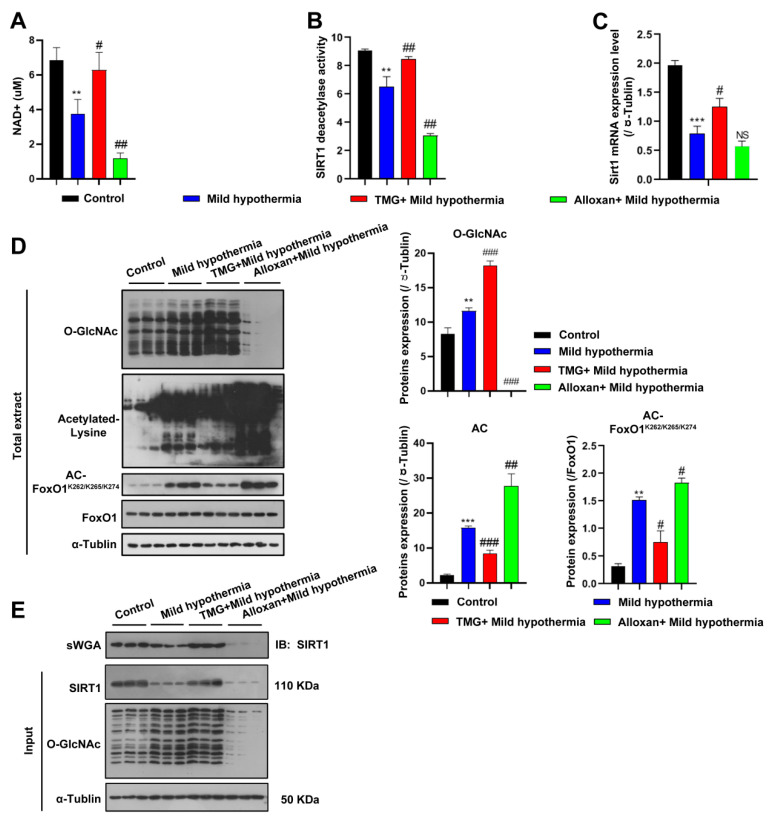
MHT increased acetylation through SIRT1 inhibition. (**A**–**C**) After 3 h of MHT at 32 °C, the C2C12 cells were collected to detect: (**A**) NAD^+^ content, (**B**) SIRT1 activity, and (**C**) *Sirt*1 mRNA expression. (**D**) O-GlcNAcylation, acetyl-Lys, and AC-FoxO1^K262/k265/k274^ protein expression levels. (**E**) The C2C12 cells were collected after 3 h of MHT at 32 °C, and the total extract was prepared, mixed with 30 µL of agarose succinylated wheat germ agglutinin, and incubated overnight at 4 °C. The glycosylated proteins were combined with agarose succinylated wheat germ agglutinin. The next day, the supernatant was discarded after centrifugation, washed three times with phosphate-buffered saline, mixed with loading buffer, and boiled. Then, the supernatant was collected for Western blotting analysis using antibodies against SIRT1 and O-GlcNAc. All results contain 3 replicates per group (n = 3/group). Data are presented as means ± sd and were analyzed by one-way ANOVA. * compared to control, ** *p* < 0.01, *** *p* < 0.001; # compared to mild hypothermia group, # *p* < 0.05, ## *p* < 0.01; ### *p* < 0.001.

To address whether CS regulates SIRT1 in the same way in C2C12 cells as in vivo, NAD^+^ expression, SIRT1 deacetylase activity, and Sirt1 transcript levels were measured. Consistent with the in vivo data, all three markers were decreased by CS and further reduced by inhibition of OGT but rescued by inhibition of OGA (Figure 6A–C). Accordingly, the expression levels of acetylated proteins, including FoxO1, were significantly increased in C2C12 cells (Figure 6D). Since SIRT1 is responsive to O-GlcNAcylation in response to CS, SIRT1 was predicted to interact with OGT, which was confirmed with the use of succinylated wheat germ agglutinin (Figure 6E), consistent with a recent report [72]. The report shows that Thr^160^/Ser^161^ (T^160^/S^161^) within the exon-2 domain of SIRT1 could be modified [72]. Although other putative sites were also predicted to be glycosylated, we focused on characterization of the modification at the N-terminal T^160^/S^161^ residues, as the exon-2 domain that harbors these residues confers binding specificities of SIRT1 to various TFs [73,74]. Furthermore, this region is intrinsically disordered, suggesting a PTM-based mechanism for regulating such protein–protein interactions [74,75]. Together, these findings suggest that OGT physically modified SIRT1.

### 2.8. Overexpression of SIRT1 Rescued Mitochondrial Defects in C2C12 Cells Exposed to MHT

**Figure 7:**
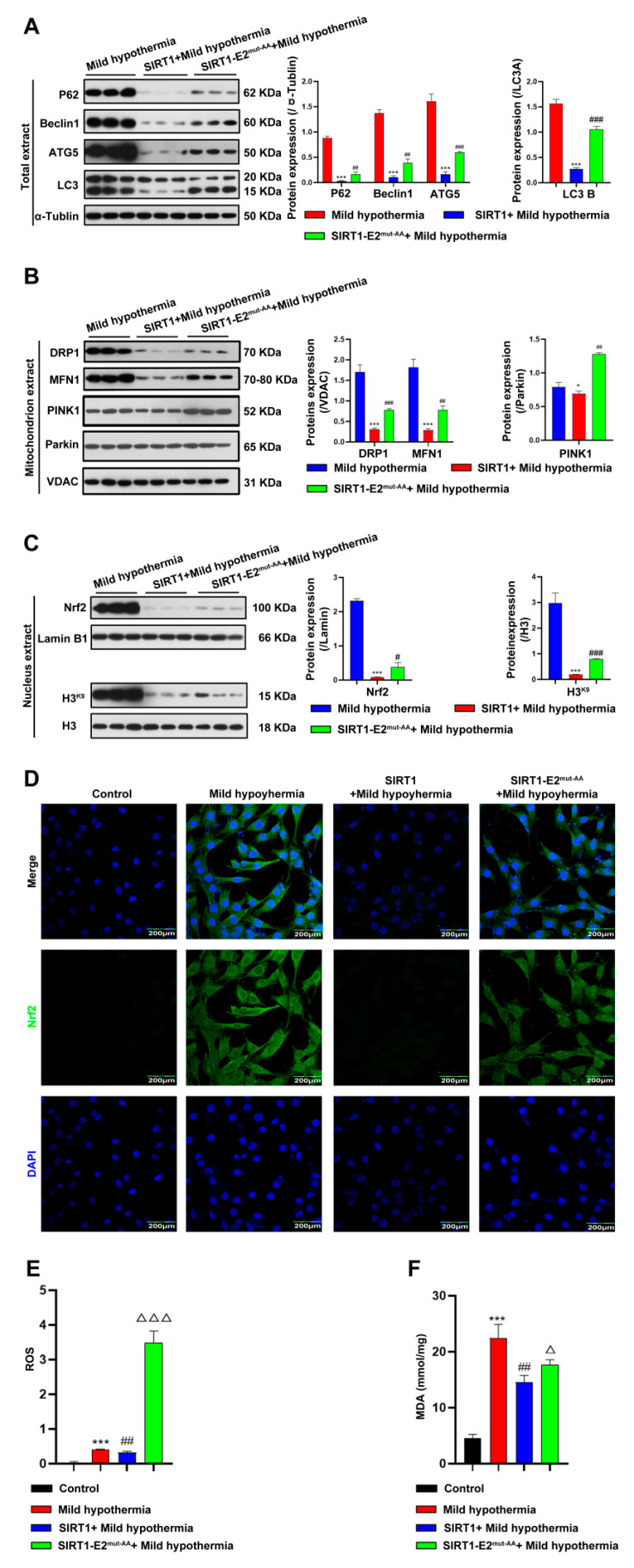
Overexpression of SIRT1 ameliorated CS-induced damage to the mitochondria. After treatment, C2C12 cells were collected and total extract, mitochondrial extract, and nuclear extract were prepared. (**A**) Autophagy-related protein expression levels in total extract. (**B**) Mitophagy-related protein expression levels in mitochondrial extract. (**C**) Nuclear extract expression level of Nrf2 and H3^K9^. (**D**) immunofluorescence staining revealed the presence of Nrf2 protein. (**E**) ROS was detected by flow cytometry. (**F**) MDA content. All results contain 3 replicates per group (n = 3/group). Data are presented as means ± sd and were analyzed by one-way ANOVA. * compared to control, * *p* < 0.05, *** *p* < 0.001; # compared to mild hypothermia group, # *p* < 0.05, ## *p* < 0.01, ### *p* < 0.001; △ compared to SIRT1+mild hypothermia group, △ *p* < 0.05, △△△ *p* < 0.001.

**Figure 8:**
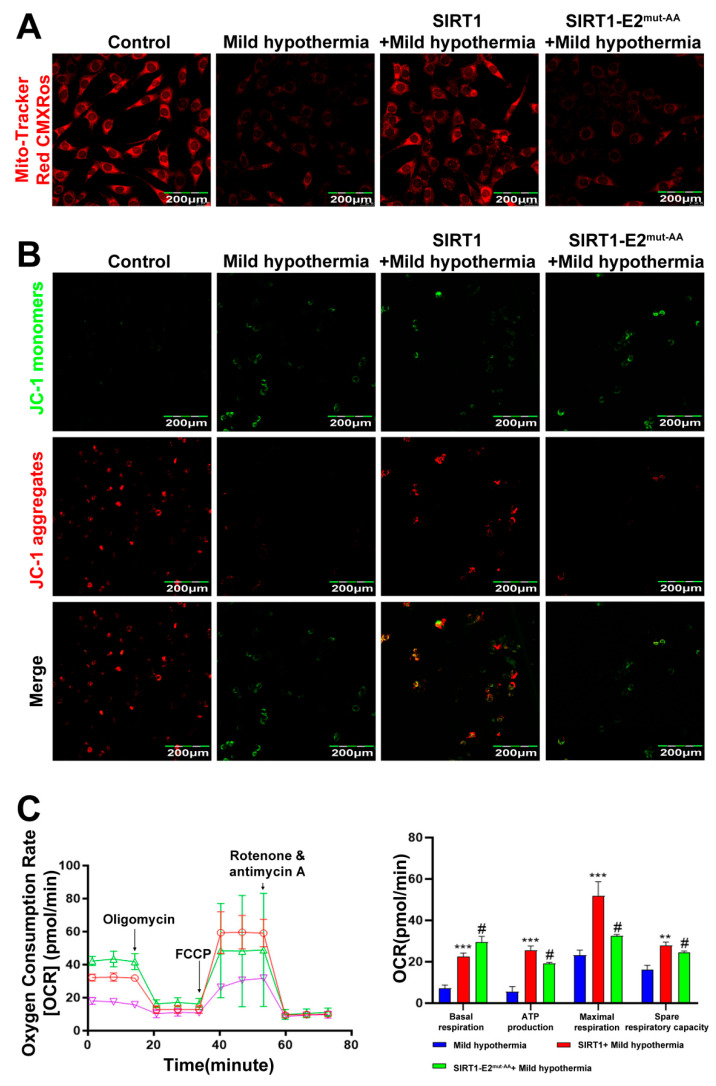
SIRT1 overexpression rescues CS-induced mitochondrial damage. After treatment, C2C12 cells were collected and stained with (**A**) MitoTracker™ Red CMXRos and (**B**) JC-1. (**C**) Mitochondrial function (i.e., basal respiration, ATP production, maximum respiration, and reserve respiratory capacity) was assessed with the cellular mitochondrial pressure test. All results contain 3 replicates per group (n = 3/group). Data are presented as means ± sd and were analyzed by one-way ANOVA. * compared to mild hypothermia group, ** *p* < 0.01, *** *p* < 0.001; # compared to SIRT1+mild hypothermia group, # *p* < 0.05.

To determine if SIRT1 is a downstream effector of OGT in response to CS, we hypothesized that glycosylation at T^160^/S^161^ in mouse SIRT1 could play a critical role in regulating cold stress-induced metabolic homeostasis imbalance. Hence, we generated the wild-type (WT) and double-site mutants (T^160^/S^161^-Ala) of SIRT1 (SIRT1-E2^mut-AA^) and assessed their functions under mild hypothermia. The results showed that overexpression of WT, but not mutant, SIRT1 reduced autophagy and mitophagy (Figure 7A,B). The results further showed that overexpression of WT SIRT1 reduced expression of Nrf2 (Figure 7C,D), as well as production of ROS and MDA (Figure 7E,F). Then, the red-fluorescent dye MitoTracker™ Red CMXRos was used to determine the abundance of mitochondria in C2C12 cells, which revealed that overexpression of WT, but not mutant, SIRT1 increased the number of mitochondria (Figure 8A). Overexpression of nonmutant WT SIRT1 improved the mitochondrial membrane potential of C2C12 cells (Figure 8B) and improved the basal respiration, ATP production, maximum respiration, and reserve respiratory capacity of C2C12 cells (Figure 8C). Together, these findings suggest that SIRT1 promoted, at least in part, the activities of OGT in the response of C2C12 cells to MHT.

## 3. Discussion

The results of the present study suggest that CS disrupts mitochondrial homeostasis, resulting in damage to SM cells. SIRT1 at residues Thr^160^ and Ser^161^ contributed to the adaptation of SM cells to CS. Notably, CS affects a variety of biochemical regulatory systems and has major impacts on thermogenesis, immune responses, and metabolism. However, the effect of CS on the metabolism of SM cells remains unclear. Although it is not surprising that pathways involved in autophagy, particularly mitophagy, are upregulated in response to acute CS, upregulation of pathways regulating mitochondrial fission and fusion suggests a compensatory mechanism for SM to ensure adequate energy production due to damaged mitochondria.

OGT is a unique enzyme that catalyzes the addition of GlcNAc to target proteins and is critical for the regulation of various cellular processes, including circadian regulation of gene expression, CS-induced thermogenesis, and gluconeogenesis. CS or β-adrenergic stimulation activates PERK, which phosphorylates OGT and subsequent glycosylation of TOM70 at Ser^94^, thereby enhancing importation of MIC19 into the mitochondria, which promotes cristae formation and respiration [76]. OGT also regulates the maintenance of hematopoietic stem cells via PINK1-dependent mitophagy [77]. Previous studies have established that OGT partially regulates mitochondrial function and mitophagy in response to CS. Of note, OGT, as a nutrient sensor, together with OGA, is also upregulated upon exposure to CS. Considering the sensory role of OGT in the modulation of cellular activities determined by nutrient availability, enhanced OGT expression and subsequent O-GlcNAcylation of its substrates serve as a protective mechanism for SM cells to mobilize metabolic and energy resources in response to CS. The results of this study indicate that mKO of *Ogt* exacerbated CS in SM cells. In SM, as in other metabolically active tissues, such as liver and adipose tissues, the nutrient sensor OGT alters the function and/or localization of thousands of substrates via O-GlcNAcylation. Although the mechanism underlying the interactions of OGT and associated substrates help cells adapt to CS, it is clear that OGT plays an instrumental, rather than detrimental, role in this pathological process. To further support this argument, inhibition of OGA activity by the specific inhibitor thiamet G ameliorated the effects of CS in SM cells.

From a mechanistic viewpoint, OGT functions through the substrate SIRT1 to regulate cellular responses to CS. There are three lines of evidence to support this hypothesis. First, SIRT1 expression and activity were downregulated in response to CS both in vivo and in vitro, leading to dramatically increased cellular acetylation. SIRT1 is an NAD^+^-dependent deacetylase that plays a key role in a wide range of biological events, including metabolism, immune responses, and aging [78]. The results of this study showed that FoxO1 expression and acetylation were increased in response to CS, accompanied by a downregulation of SIRT1 expression and activity. Because FoxO1 is involved in autophagy and mitophagy [79,80], it is reasonable to speculate that SM responds to CS by increasing the expression of OGT, which glycosylates and thus stabilizes SIRT1. O-GlcNAcylated SIRT1 then deacetylates FoxO1 and downregulates autophagy and mitophagy. This argument is supported by the fact that MHT-induced inhibition of OGA led to decreased expression and acetylation of FoxO1, whereas inhibition of OGT had the opposite effect. Since SIRT1 can deacetylate a broad range of substrates ranging from members of the histone family, such as H1K^26^, H3K^9^, and H4K^16^, proteins related to DNA damage repair (e.g., NBS1 and Ku70), gluconeogenesis (e.g., CRTC2), and immune response (e.g., NF-κB and FOXP3) [81], it was difficult to identify exactly which substrates are responsible for the observed actions of SIRT1 on autophagy and mitophagy in response to CS. For example, in addition to FoxO1, SIRT1 is reported to regulate autophagy and mitophagy through PGC1 and Mfn2 [82,83]. Regardless, FoxO1 promotes, at least in part, the activities of SIRT1 in CS-induced autophagy and mitophagy. Furthermore, OGT adds a sugar derivative to SIRT1 to either increase stability or protect against degradation. Since protein acetylation can activate or silence the expression of various genes and alter the activities and locations of numerous enzymes, it is especially important to adjust this posttranslational modification. These results showed that CS can induce protein acetylation while concomitantly downregulating SIRT1 expression. It is possible that downregulation of SIRT1, along with other deacetylases, is responsible for enhanced acetylation in response to CS. Second, this study demonstrated the physical interaction between OGT and SIRT1 by enriching O-GlcNAcylation proteins using succinylated wheat germ agglutinin and detection of SIRT1 expression (Figure 6E). Third, residues T^160^ and S^161^ of SIRT1 are sites of O-GlcNAcylation [72]. Together, these findings further demonstrate the protective effect of SIRT1 O-GlcNAcylation at residues T^160^ and S^161^ in SM cells in response to CS.

The mitochondria are the main sites of aerobic respiration for energy production and metabolism. Disrupted homeostasis of the intracellular environment and mitochondrial damage can release apoptosis-related proteins and produce a series of reactions resulting in apoptosis [84]. Numerous studies have shown that external stimuli can significantly downregulate the functions of mitochondria, such as basal respiration, ATP production, maximal respiration, and reserve respiratory capacity [85,86,87]. The results of this study showed that basal respiration, ATP production, maximum respiration, and reserve respiration capacity were significantly downregulated in C2C12 cells exposed to MHT, consistent with previous reports. However, overexpression of SIRT1 significantly upregulated these indicators, while deglycosylation of SIRT1 at residues T^160^ and S^161^ did not improve mitochondrial function. Mitochondrial membrane potential is a key indicator of mitochondrial health [88]. X-rosamine (chloromethyl-X-rosamine, CMXRos) is a cell permeable derivative of X-rosamine to specifically label bioactive mitochondria in cells. Detection of mitochondrial membrane potential was conducted as described previously [89]. The results of the present study showed that the mitochondrial membrane potential of C2C12 cells was significantly decreased after MHT treatment, while overexpression of SIRT1 had the opposite effect. However, the membrane potential of SIRT1 was not restored after deletion of O-GlcNAcylation at residues T^160^ and S^161^, consistent with the results obtained using JC-1. These results indicate that MHT damaged mitochondrial function in C2C12 cells. ROS is an intermediate product of mitochondrial aerobic respiration, and small amounts of ROS exist in cells under normal physiological conditions. However, damaged mitochondria produce large amounts of ROS, resulting in disrupted homeostasis of the intracellular environment [90]. MHT significantly decreased the mitochondrial membrane potential of C2C12 cells, and a large amount of ROS entered the cytoplasm via the mitochondria, which damaged other organelles due to OS. The results of transmission electron microscopy showed that the mitochondrial ridges of C2C12 cells disappeared after exposure to MHT along with morphological and structural damage. In addition, Western blotting analysis detected the upregulation of proteins associated with autophagy and mitophagy. Overexpression of SIRT1 alleviated autophagy and mitophagy in C2C12 cells exposed to MHT. However, deletion of O-GlcNAcylation of SIRT1 at residues T^160^ and S^161^ did not improve MHT-induced autophagy and mitophagy or downregulate ROS production. MDA is a product of lipid oxidation that is commonly used as a biomarker of OS [91]. The results showed that the production of MDA in C2C12 cells was consistent with that of ROS, suggesting that MHT massively increased ROS production and accumulation of MDA, resulting in OS in cells. Nrf2 is an important OS-induced transcription factor and an important regulator of the intracellular redox state. Under normal physiological conditions, Nrf2 is located in the cytoplasm, but enters the nucleus in response to the actions of ROS and activates transcription of downstream target genes to enhance antioxidant activity. The results of Western blotting and immunofluorescence analyses showed that Nrf2 expression was significantly up-regulated in the nuclei of C2C12 cells after exposure to MHT, and acetylation of the ninth amino acid residue of histone H3 was also significantly upregulated. However, deletion of the O-GlcNAc moiety from residues T^160^ and S^161^ of SIRT1 had no significant effect. Overall, the results of this study demonstrated that CS induced the accumulation of ROS and MDA in mouse SM cells, resulting in OS and a large amount of Nrf2 entering the nucleus, thereby improving antioxidant capacity. A large amount of ROS can disrupt the structure and function of mitochondria, resulting in disruption to the intracellular environment. However, enhancement of O-GlcNAcylation of SIRT1 at residues T^160^ and S^161^ prevented damage to the mitochondria. Cellular autophagy is a cytoprotective mechanism to maintain cellular homeostasis by specifically degrading damaged or redundant organelles. Disrupted homeostasis induces autophagy to degrade and clear damaged cells to restore homeostasis. Mitochondria are important for the occurrence of autophagy. Upregulation of LC3B expression is a marker of autophagy and Beclin1, as a key protein involved in autophagy initiation, and P62, an important ubiquitin junction protein in autophagy degradation. Both play important roles in autophagy. The results of this study showed that the expression levels of autophagy-related proteins were significantly upregulated in response to CS, indicating that CS activates autophagy. However, enhanced O-GlcNAcylation of SIRT1 at residues T^160^ and S^161^ slowed down autophagy. The results of transmission electron microscopy also demonstrated that CS induced autophagy and morphological damage to the mitochondria. PINK1 is a Ser/Thr kinase that can pass through the mitochondrial membrane. The membrane potential of damaged mitochondria is decreased, as is the ability to degrade PINK1, which leads to the accumulation of PINK1 on the outer mitochondrial membrane, thereby increasing the recruitment of Parkin and facilitating ubiquitination and degradation of damaged mitochondria. The results showed that the expression levels of PINK1 and Parkin, as markers of mitophagy in C2C12 cells, were significantly upregulated after exposure to MHT. However, enhanced O-GlcNAcylation of SIRT1 at residues T^160^ and S^161^ blocked the recruitment of Parkin by PINK1, thereby slowing the initiation of mitophagy.

The results of this study indicate that SIRT1 expression was inhibited, and deacetylation was reduced in mouse C2C12 cells exposed to CS. Meanwhile, activation of the SIRT1-Foxo1 pathway leads to increased histone acetylation, OS, and recruitment of Nrf2 into the nucleus, resulting in structural and functional damage to the mitochondria and excessive autophagy and mitophagy in mouse C2C12 cells. In addition, O-GlcNAcylation of SIRT1 at residues T^160^ and S^161^ alleviated CS-induced dysregulation of mitochondrial homeostasis in mouse SM cells as a protective mechanism. In conclusion, O-GlcNAcylation of SIRT1 contributes to adaptation of SM cells to CS, thereby providing an important target to further elucidate the underlying mechanisms. Nonetheless, further studies are warranted to clarify the role of O-GlcNAcylation of SIRT1 in the protection of SM cells from autophagy and mitophagy, and to identify molecular targets for the management of MHT. Although this study highlighted the importance of O-GlcNAcylation of SIRT1 in the adaptation of SM to CS, other mechanisms cannot be ruled out. For example, many mitochondrial proteins involved in the tricarboxylic acid cycle and electron transport chain are O-GlcNAcylated. Therefore, it is conceivable that these proteins would be hyperglycosylated in response to CS. Hence, further investigation is warranted to clarify the functions of hyperglycosylated proteins in the maintenance of mitochondrial integrity. In addition, this study also has shortcomings. Skeletal muscle is mainly composed of muscle cells (myotubes) rather than myoblasts, while C2C12 are not myotubes. Therefore, this study also has some limitations, and we will improve it in future research.

## 4. Materials and Methods

### 4.1. Experiments with Mice

In all mouse experiments, 6-week-old male mice were used in the experiments. *Ogt*^LoxP/+^ mice were purchased from the Jackson Laboratory (JaxMice, strain#: 004860, allele symbol: B6.129-*Ogt^tm1Gwh^*/J). *Ogt* gene knockout was achieved by breeding *HSA*^Cre/+^ (JaxMice, strain#: 006149, allele symbol: B6.Cg-Tg (ACTA1-cre) 79Jme/J) males with *Ogt*^LoxP/LoxP^ females to generate *HSA*^+/+^; *Ogt*^LoxP/Y^ (WT); and *HSA*^Cre/+^; *Ogt*^LoxP/Y^(*Ogt* mKO) mice. Mice were fed in individually ventilated cages (IVC) at an ambient temperature of 26 °C ± 2 °C with free access food and water on a 12 h light/dark cycle. For cold stress experiments, mice were exposed to 4 °C for 3 h a day for a period of one week. All animal procedures were approved and conducted in accordance with the guidelines set by the Heilongjiang Bayi Agricultural University Animal Care and Use Committee.

### 4.2. Histological Staining

Muscles were isolated and fixed with 10% formalin for 24 h, then made into tissue sections. Masson trichrome staining was performed according to the manufacturer’s instructions (Solarbio Life Sciences, G1346, Beijing, China). The β-Galactosidase staining kit was purchased from Beyotime Biotechnology (C0602) (Shanghai, China), and the staining was performed according to the manufacturer’s instructions. Images were taken using a fluorescent microscope (high resolution slide scanning system, Pannoramic MIDI, 3DHISTECH Ltd., Budapest, Hungary).

### 4.3. Western Blotting

Muscle tissues or C2C12 cells were lysed in ice-cold RIPA Lysis Buffer (Beyotime biotechnology, P0013B) containing 1% protease and PMSF (Beyotime biotechnology, ST506) on ice. After the collection of lysate removed debris by centrifugation, protein concentrations were measured by the BCA assay (Beyotime biotechnology, P0010S). Mitochondrial extract and nuclear extract from muscle tissues or C2C12 cells using the Mitochondria Isolation Kit (Beyotime Biotechnology, C3606) and Nuclear and Cytoplasmic Protein Extraction Kit (Beyotime Biotechnology, P0028). Protein samples were mixed with SDS-PAGE Sample Loading Buffer (Beyotime Biotechnology, P0015) and incubated at 70 °C for 10 min. Proteins were transferred to 0.45 μm PVDF membranes (Merck Millipore, IPVH0010), and blocked with 5% skim milk in 10 mM Tris TBS-0.1% Tween 20 (TBST) for at least 1 h. Primary and HRP conjugated secondary antibodies were diluted in 5% skim milk in TBST. The HRP substrate ECL (Merck Millipore, WBKLS0500) was used to detect signals. Band intensities were quantified by Image-Pro Plus. The antibodies used for Western blotting analysis were as follows: MGEA5/OGA antibody (Abcam, ab124807); OGT/O-Linked N-Acetylglucosamine Transferase (Abcam, ab96718); O-GlcNAc antibody (CTD110.6, CST, 9875); SIRT1 polyclonal antibody (Proteintech, 1:1000, 13161-1-AP, Wuhan, China); FoxO1 polyclonal antibody (Proteintech, 1:1000, 18592-1-AP); AC-FoxO1 (Abclonal, 1:1000, 3560748323); acetylated-lysine antibody (CST, 1:1000, 9441); P62/SQSTM1 polyclonal antibody (Proteintech, 1:1000, 18420-1-AP); Beclin1 polyclonal antibody (Proteintech, 1:1000, 11306-1-AP); autophagy protein 5 (ATG5) polyclonal antibody (Proteintech, 1:1000, 10181-2-AP); microtubule-associated protein light chain 3 (LC3) polyclonal antibody (Proteintech, 1:1000, 14600-1-AP); DRP1 (C-terminal) polyclonal antibody (Proteintech, 1:1000, 12957-1-AP); MFN1 polyclonal antibody (Proteintech, 1:1000, 13798-1-AP); mutations in the PTEN-induced kinase 1 (PINK1) polyclonal antibody (Proteintech, 1:1000, 23274-1-AP); PARK2/Parkin polyclonal antibody (Proteintech, 1:1000, 14060-1-AP); nuclear factor E2-related factor 2 (Nrf2) rabbit polyclonal antibody (ABclonal, 1:1000, A1244); histone-H3 polyclonal antibody (Proteintech, 1:1000, 17168-1-AP); acetyl-histone H3 (Lys9) (C5B11) rabbit monoclonal antibody (CST, 1:1000, 9649); VDAC polyclonal antibody (Proteintech, 1:1000, 10866-1-AP); Lmin B1 polyclonal antibody (Proteintech, 1:2000, 12987-1-AP); alpha tubulin monoclonal antibody (Proteintech, 1:20,000, 66031-1-Ig); HRP-conjugated Affinipure goat anti-mouse IgG (H+L) (Proteintech, 1:10,000, SA00001-1); HRP-conjugated affinipure goat anti-rabbit IgG (H+L) (Proteintech, 1:10,000, A00001-2).

### 4.4. Detection of Reactive Oxygen Species (ROS)

Mouse skeletal muscles were quickly frozen, cut to a thickness of 8 µm, and placed on glass slides. Fresh dihydroethidium (DHE) solution (Beyotime Biotechnology, S0063) was applied to each tissue section, and the section was incubated for 30 min at 37 °C in the dark. Fluorescent images were captured using a fluorescent microscope (high resolution slide scanning system, Pannoramic MIDI, 3DHISTECH Ltd., Budapest, Hungary). After mild hypothermia treatment (32 °C), C2C12 cells were stained using a ROS assay kit following the manufacturer’s instructions (Beyotime, Reactive Oxygen Species Assay Kit, S0033S, Shanghai, China). The cells were then analyzed using a flow cytometer (Beckman, CytoFLEX FCM, Shanghai, China).

### 4.5. Malondialdehyde (MDA) Assay

Muscle tissues or C2C12 cells were lysed in ice-cold RIPA Lysis Buffer (Beyotime biotechnology, P0013B) containing 1% protease and PMSF (Beyotime biotechnology, ST506) on ice. After collection of lysate removed debris by centrifugation, protein concentrations were measured by the BCA assay (Beyotime biotechnology, P0010S). Protein concentration was determined using the Enhanced BCA Protein Assay Kit (Beyotime, P0010S). The Lipid Peroxidation MDA Assay Kit (Beyotime, S0131) was used to measure lipid peroxidation at 532 nm using a microplate reader (Mindray, MR-96A, Shenzhen, China).

### 4.6. Cell Mitochondrial Stress Examination

Fresh muscle tissue was washed in DMEM, cut, and placed in the islet capture plate. C2C12 cells were seeded into XF cell culture microplates. Before the experiments, the probe plate containing the XF calibrant was cultured overnight in a carbon dioxide-free incubator to achieve balance. During the experiment, the medium was changed to XF test medium supplemented with 5 mM sodium pyruvate, 10 mM glucose, and 2 mM glutamine, and balanced in a non-CO_2_ incubator for 1 h. Oxygen consumption rate (OCR) was monitored by sequential injections of 1.5 μM oligomycin, 1 μM FCCP, and 0.5 μM rotenone/antimycin A (Seahorse XF Cell Mito Stress Test Kit, Agilent, Santa Clara, CA, USA), according to the manufacturer’s instructions.

### 4.7. Measurement of NAD^+^

Fresh muscle tissue or C2C12 cells were collected and rinsed with ice-cold PBS. The NAD^+^ and NADH levels of muscle tissue and C2C12 cells were measured at 450 nm with a microplate analyzer using the NAD^+^/NADH Assay Kit with WST-8 (Beyotime, S0175). The following formula was used to derive NAD^+^: NAD^+^ = NAD_total_ − NADH.

### 4.8. SIRT1 Activity Determination

Fresh muscle tissue or C2C12 cells were collected, rinsed with ice-cold PBS, and nuclear protein was extracted using a nuclear and cytoplasmic protein extraction kit (Beyotime, P0027). SIRT1 activity was measured using a commercial kit following the manufacturer’s instructions (Sigma, CS1040, Shanghai, China). Fluorescence intensity was detected by a fluorescence microplate reader, and the activity of SIRT1 was calculated. Excitation = 340–380 nm, Emission = 430–460 nm.

### 4.9. Glycosylation Detection

Muscle tissue or C2C12 cells were lysed using the NP-40 Lysis Buffer (Beyotime, P0013F). Protein concentration was determined using the Enhanced BCA Protein Assay Kit (Beyotime, P0010S). Thirty μL of agarose succinylated wheat germ agglutinin (Vectorlabs, AL-1023S) was added to 500 μg total lysate. The mixture was then incubated overnight at 4 °C, centrifuged for 2 min at 3500 rpm the next day, and the supernatant was discarded. The beads were then washed 3 times with NP-40 Lysis Buffer (Beyotime, P0013F) and the supernatant was discarded. The SDS-PAGE Sample Loading Buffer (Beyotime, P0015L) was added to the beads and boiled for 5 min. The supernatant was collected and used for Western blotting analysis.

### 4.10. Mitochondrial Imaging

When C2C12 cells reached 70% confluence, the culture medium was removed and the working solution of MitoTracker Red CMXRos (Beyotime, C1049) was added to the cells. After an incubation at 37 °C for 30 min, the medium was changed to fresh culture medium pre-warmed at 37 °C. Images were taken using a laser scanning confocal microscope (Leica, TCS-SP2, Weztlar, Germany).

### 4.11. JC-1 Imaging of Mitochondrial Membrane Potential

When C2C12 cells reached 70% confluence, the medium was removed, and the cells were washed once with PBS. Next, 1 mL of the working fluid JC-1 (Beyotime, C2006) was added to the cells according to the manufacturer’s instructions. Images were taken using a laser scanning confocal microscope (Leica, TCS-SP2, Germany).

### 4.12. Immunofluorescence

C2C12 cells were fixed with 4% paraformaldehyde, permeabilized with 0.3% Triton X-100, and blocked with 3% bovine serum albumin. Cells were then incubated in the primary antibody solutions overnight at 4 °C. Sections were washed in 1 × PBS three times for 5 min each after being incubation in the primary antibody overnight at 4 °C. The cell craws were then incubated in Alexa Fluor coraLite488-conjugated Affinipure Goat Anti-Mouse IgG (Proteintech, SA00013-1) or Alexa Fluor coraLite594-conjugated Goat Anti-Rabbit IgG (Proteintech, SA00013-4) highly cross-absorbed antibodies, respectively, for 1 h at room temperature, washed in PBS, and mounted in fluorescent mounting medium for microscopy. Images were taken using a laser scanning confocal microscope (Leica, TCS-SP2, Germany).

### 4.13. Statistical Analysis

Statistical analyses were performed by Graphpad Prism 8.0.1 software (Graphpad Software, San Diego, CA, USA). Methods of statistical analyses were chosen on the basis of the design of each experiment and are indicated in the figure captions. The data were presented as means ± sd. A value of *p* ≤ 0.05 was considered statistically significant.

## Data Availability

The datasets generated in this study are available upon reasonable request from the corresponding author.

## Supplementary Materials

The following are available online at https://www.mdpi.com/article/10.3390/ijms232314520/s1.
