# Supplementary material for: O-GlcNAcylation of SIRT1 Protects against Cold Stress-Induced Skeletal Muscle Damage via Amelioration of Mitochondrial Homeostasis
Source: Int J Mol Sci. 2022 Nov 22;23(23):14520. doi: 10.3390/ijms232314520 (PMC9737900; doi:10.3390/ijms232314520)
Supplement: Supplementary file 1 [file ijms-23-14520-s001.zip › ijms-2022119-supplementary.pdf]

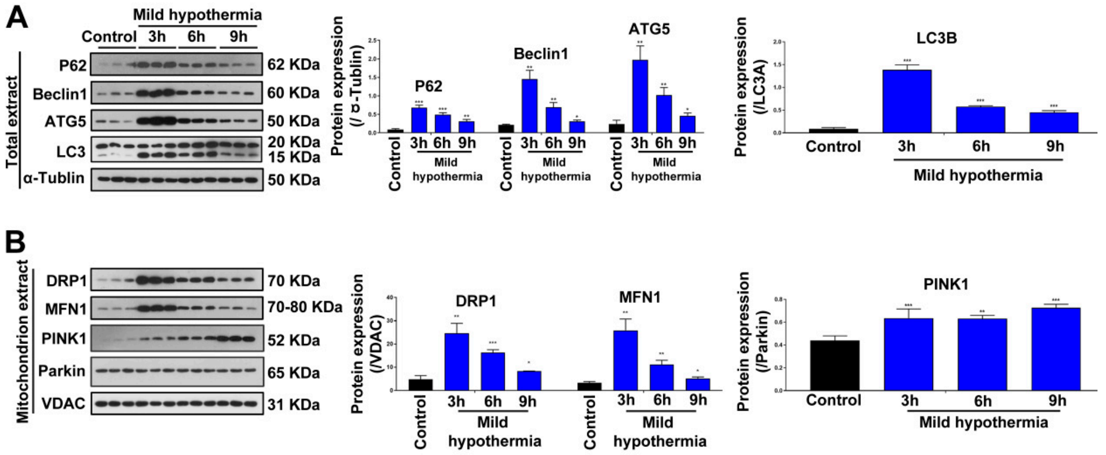

Supplementary Figure S1. Statistics of autophagy and mitophagy-related proteins in C2C12 cells after exposure to MHT for different durations. C2C12 cells were cultured at 32 °C for 3, 6, and 9 h, then collected to prepare total extract and mitochondrial extract. The expression levels of autophagy and mitophagy-related proteins in C2C12 cells were detected by western blot analysis. n = 3/group. Data is presented as the mean ± SD (one-way ANOVA). *$p < 0.05$; **$p < 0.01$; ***$p < 0.001$ vs. the control group.
